# The Impact of Sibling Relationships on Behavioral and Sexual Health among Latino Sexual Minority Men

**DOI:** 10.1007/s10508-024-02832-6

**Published:** 2024-03-29

**Authors:** Juan Pablo Zapata, Edwin Rojas, Petra Durán, Angel J. Martínez, Homero E. del Pino

**Affiliations:** 1 Institute for Sexual and Gender Minority Health and Wellbeing, Feinberg School of Medicine, Northwestern University, Chicago, IL USA; 2 St. John’s Community Health, Los Angeles, CA USA; 3 Psychiatry and Human Behaviors, Charles R. Drew University of Medicine and Science, Los Angeles, CA 90059 USA; 4 Psychiatry and Behavioral Sciences, General Internal Medicine, David Geffen School of Medicine at UCLA, Los Angeles, CA USA; 5 Geriatric Research, Education, and Clinic Center, VA Greater Los Angeles Healthcare System (Geriatric Research, Education, and Clinical Center), Los Angeles, CA USA; 6 Charles R. Drew University of Medicine and Science, Los Angeles, CA USA

**Keywords:** HIV, PrEP, Latino sexual minority men, Family intervention, Sexual orientation

## Abstract

Pre-exposure prophylaxis (PrEP) is a highly effective method for preventing HIV acquisition and plays a crucial role in the *Ending the HIV Epidemic in the US* initiative. However, there are various barriers that hinder the access and uptake of PrEP among Latino sexual minority men (SMM) at individual, interpersonal, and cultural levels. While the significance of cultural factors in designing and implementing HIV prevention programs for Latino populations has been consistently emphasized in the literature and prioritized by the Centers for Disease Control and Prevention, few studies have directly integrated these cultural factors into their programs. Our study aimed to investigate the potential influence of siblings in promoting the utilization of PrEP for HIV prevention, an area that has received limited attention. We conducted interviews with 31 pairs of siblings between December 2020 and January 2021, which were held in either English or Spanish and lasted approximately 45–60 min. The data were analyzed using a deductive thematic content analysis approach. The interviews revealed several key themes and categories, including: (1) Sibling support for coming out; (2) Types of support that siblings provide to each other for behavior change; (3) Sibling support for PrEP; and (4) The impact of the study interview on the quality of the sibling relationships. Our findings indicated that siblings were willing to provide support for PrEP in various ways, ranging from emotional support for brothers who may be concerned about potential rejection to practical support such as transportation or financial assistance. These results have significant implications for the design of HIV prevention interventions for Latinos. Incorporating siblings or other extended family members into these interventions can facilitate communication between siblings and their brothers, ultimately encouraging the use of PrEP or similar prevention methods. By considering the unique dynamics and support systems within Latino communities, researchers can develop more effective strategies to promote HIV prevention and support the well-being of Latino SMM.

## Introduction

Since the early days of the HIV epidemic, there has been a disproportionate impact on Latino sexual minority men (SMM, i.e., gay, bisexual, and other non-heterosexual identified men). While there has been a slight decrease in the number of new cases for non-Hispanic White SMM, with 9000 cases in 2010 dropping to 8900 in 2019, the rates of new HIV cases among Latino SMM have increased. In 2010, there were 6800 new cases, but by 2019 that number had risen to 7900 (CDC, 2021a, 2021b). This increase is particularly significant considering that Latinos represent one of the largest and fastest growing ethnically diverse groups in the USA. In 2020, they accounted for 19% of the population and were responsible for over half of the country’s population growth from 2010 to 2020 (Pew Research Center, 2022)

Studies have demonstrated that Latino SMM face barriers to sexual health education due to discrimination and stigma surrounding their ethnicity, sexual orientation, race, and immigration status (de Castro et al., 2018; Martinez et al., 2011; Rhodes et al., 2006). These barriers have contributed to disparities in HIV prevention between Latino SMM and their White counterparts. These disparities are especially pronounced when it comes to pre-exposure prophylaxis (PrEP) use, with only 21% of Latino PrEP candidates being prescribed PrEP compared to 78% of non-Hispanic White PrEP candidates (Kamitani et al., 2020). Several studies have identified the following challenges faced by Latino SMM in accessing PrEP: medical mistrust, lack of familial social support, limited communication about sexual health with healthcare providers, stigma, and socioeconomic burdens (Zapata et al., 2023).

Families play a crucial role in shaping the sexual health education of adolescents and young adults. As the closest and most fundamental social system, they have a significant impact on their development (Miller, 2002; Morris & Rushwan, 2015; Ryan et al., 2010). Positive family relationships, effective communication about sexuality and safer sexual behaviors, academic support, and monitoring peer activities are key factors in promoting healthier sexual development (Ryan et al., 2010). Not surprisingly, most research that involves families has focused on the role of parents or primary care takers in sexual health intervention efforts as opposed to other familial members such as siblings and/or cousins (Wight & Fullerton, 2013). Studies indicate that parent–adolescent communication about various topics is linked to delayed onset of sexual behavior and reduced risk of HIV transmission among young people who discuss sex with their parents (Miller et al., 1999; Rogers, 2017). In regard to HIV prevention, Latino parents may have difficulty accepting and/or understanding the risks associated with SMM (Deutsch & Crockett, 2016). This, in turn, can lead to lack of communication between family members about sexual orientation and potentially unsafe behaviors. Moreover, cultural differences stemming from a traditional masculine gender role can prevent fathers from engaging in meaningful discussions about sexuality or risk reduction techniques (Abreu et al., 2020; Glass & Owen, 2010; Mogro-Wilson & Cifuentes, 2021). Thus, comprehending how other family members can assist in HIV prevention among Latino SMM is critical, particularly among families who may hold stronger cultural ties to their country of origin (Lescano et al., 2009).

One approach to supporting HIV prevention among Latino SMM is through sibling relationships, yet there is a dearth of research on siblings compared to adolescent–parent or peer relationships (Cardoza et al., 2012; Grossman et al., 2015, 2020; Stauss et al., 2011; Whiteman et al., 2014). Research on younger siblings has indicated that having an older sibling often contributes to an increase in risky behaviors, such as early sexual initiation and substance use (Coleman-Minahan & Scandlyn, 2017). However, the factors that influence this relationship, including gender, sibling relationship quality, and other family dynamics, are still largely speculative (Averett et al., 2011; McHale et al., 2009). It is important to note that the influence of older siblings on the sexual behaviors of their younger siblings may differ between Latino families and non-Hispanic White families due to their distinct socioeconomic and cultural contexts (Long et al., 2022). On the other hand, positive sibling relationships characterized by warmth and acceptance have been found to enhance social competence, self-esteem, and self-regulation during adolescence (Padilla-Walker et al., 2010). Additional research has also found that positive sibling relationship qualities serve as protective factors and buffer the influence of stressors such as family conflict (Gass et al., 2007), low parent support (Fry et al., 2021), and poor peer relationships (Milevsky & Levitt, 2005; Toomey & Richardson, 2009). Few studies, however, have examined the ways in which sibling relationships can support and/or hinder HIV prevention efforts among Latino SMM.

To our knowledge, only a few studies looked at the influence of Latino sibling relationships on adolescents’ sexual health behavior and the mechanisms in which older siblings influence this behavior (Cardoza et al., 2012; Eversole et al., 2017; Malacane & Beckmeyer, 2016). Coleman-Minahan and Scandlyn (2017) found that the challenges and difficulties of the immigrant experience—such as poverty, family separation, and discrimination—strengthen the positive and protective qualities of older siblings. As a result, older sisters, in particular, often take on the responsibility of caring for their younger siblings. Additional studies have supported this finding, indicating that siblings of Mexican-origin tend to have positive and supportive relationships (Killoren et al., 2015; Updegraff et al., 2005). Moreover, siblings may buffer the negative effects of family instability or low socioeconomic status (East & Khoo, 2005; McHale et al., 2009), which are known risk factors for HIV risk (CDC, 2022). Consistent with prior research, Coleman-Minahan and Scandlyn (2017) also found that older siblings provide sexual health advice to protect younger siblings from risky sexual behaviors (Killoren & Roach, 2014). However, these findings are restricted to sister–sister dyads in Latino families and have not yet been explored in mixed-gender dyads or among Latino SMM. Given the unique cultural processes of Latino families and the additional stress caused by the sexual minority status of SMM, it is crucial to investigate the impact of siblings on the sexual and reproductive health behaviors of Latino SMM (Cerezo et al., 2020). This matters because HIV prevention strategies continue to overlook the siblings of Latino SMM, even though Latino SMM have reported that social support from their siblings is an important part of their lives and that support and concern from their siblings could influence their HIV risk behaviors (Garcia et al., 2022).

### Current Study

We conducted interviews with sibling dyads (i.e., brother–Latino SMM or sister–Latino SMM) to explore whether siblings can be engaged in PrEP uptake strategies for HIV prevention. Understanding the dynamics of these relationships can lead to the development of effective and culturally appropriate interventions and support systems that address the unique needs of Latino SMM.

We used a mixed methods design (quantitative surveys and dyadic qualitative interviews) to explore the relationship dynamic between Latino SMM and their siblings within the context of sexual health and HIV prevention.

## Method

### Participants

Latino SMM had to (1) self-identify as Latino, (2) be assigned male (sex) at birth, (3) be aged 18–39 years, (4) report an HIV-negative status, (5) be willing to talk about sexual health and PrEP use with a sibling (no gender requirement), (6) report never having used PrEP, (7) report sex with a male in the past 6 months, and (8) meet the PrEP eligibility criteria set by the CDC (e.g., report condomless anal sex in the past 6 months) (CDC, 2021b). Siblings had to (1) be ≥ 18 years old, (2) report a close relationship with their Latino SMM brother, and (3) be willing to talk about sexual health and PrEP use with their Latino SMM brother.

We used convenience sampling to recruit participants. Before COVID-19 restrictions, we conducted outreach at traditional venues (e.g., community clinics, community events, and social venues throughout Los Angeles County). After COVID-19 restrictions went into effect, we transitioned to online recruitment: Facebook, Twitter, Instagram, and Grindr.

### Procedure

We interviewed a sample of PrEP-naïve Latino SMM together with a sibling that they trusted to participate in a dyadic interview. Interviews were conducted by one or more of the three members of the research the team, including the PI. All three identify as Latinx, two as gay men and one as a heterosexual cisgender woman. We used open-ended questions to allow participants to describe their experiences in their own words (Creswell & Creswell, 2017; Creswell et al., 2004). This also allowed the interviewer to clarify participants’ responses and to observe the dynamic between siblings, such as how expressive and comfortable they were talking about sexual health. Participants were compensated $50. The first three dyadic interviews were conducted in-person in Los Angeles in early March 2020, before COVID-19 restrictions were enacted. The remaining 28 interviews were conducted and recorded on Zoom and completed by August 2020.

### Measures

Participants, including Latino SMM and their siblings, were asked to provide information such as their age, place of birth, educational status, and living situation. To assess the risk of HIV infection, Latino SMM were also asked to rate their perceived lifetime risk on a 5-point Likert scale. Additionally, they were queried about their awareness of PrEP, access to PrEP, willingness to use PrEP, and whether they would consider using PrEP to alleviate their siblings’ concerns about HIV infection, using a similar 5-point Likert scale. The response options ranged from strongly disagree to strongly agree. Siblings who were enrolled in the study were asked similar questions. These questions covered a range of topics including their level of concern for their brother contracting HIV, whether they believed their brother knew how to access PrEP, if their brother’s decision to use PrEP would alleviate their concerns about his HIV status, and specific ways in which they would support their brother’s decision to use PrEP. The response options for each question followed a consistent 5-point Likert scale.

Our research team collaborated with providers and community partners to develop the interview guide. Initially, our team conducted a literature review to draft a comprehensive list of potential topics. To operationalize the research question, we created a grid using information-motivation-behavioral (IMB) model constructs as rows and the first three stages of change from the Transtheoretical Model of Behavior Change as the columns. For example, the “information” row contained columns for pre-contemplation, contemplation, and preparation. For more information, see del Pino et al. (2023). After refining the questions, the community partners were provided with the final version of the interview guide and offered additional input before the study commenced. These questions covered various aspects of their relationship, including the ways in which Latino SMM and their siblings support each other and their family dynamics (“Can you share about a time when you helped each other change a behavior, like exercising more?”). Additionally, the interviews explored topics such as Latino SMM experiences of coming out to their siblings (“How did you react when you found out that your brother was gay?”) and the dynamics of their relationships. Similar questions were also provided to the participating siblings. Latino SMM and their siblings were also asked specific questions about PrEP, including their awareness of PrEP as a prevention strategy, reasons for considering or not considering PrEP, and the level of support siblings would provide if their brother decided to use PrEP. For siblings who were unfamiliar with PrEP, a concise overview was given, explaining its purpose, effectiveness, and various modes of administration, such as oral and injection.

### Data Analyses

A research assistant who is fluent in Spanish and English transcribed the interviews in the language they were conducted, and later translated the three interviews conducted in Spanish into English. We conducted a content analysis guided by the IMB skills model and the transtheoretical model of behavior change (Prochaska & DiClemente, 1983) using a process developed by Strauss and Corbin (1990). For more details, please refer to del Pino et al. (2023). MAXQDA software was used to code and provide organization (Kuckartz & Rädiker, 2019). At the onset of the coding process, two bilingual, bicultural research team members independently coded five transcripts to develop a preliminary codebook. Discrepancies were discussed to ensure consensus on code application, adequacy of definitions, and completeness of the codebook. Subsequently, the codebook was applied to each interview, prioritizing initial codes that held significant “analytical power” (Charmaz, 2014) in terms of frequency or significance. The phases of analysis were interconnected and supported by continuous memo writing. The two researchers double-coded transcripts until reaching inter-coder agreement 70% agreement, which was calculated on MAXQDA. We then systematically assessed the sibling relationships between self-identified heterosexual and gay siblings, as well as the relationships between older and younger participants. This allowed us to gain a deeper understanding of the dynamics of these relationships, patterns, or differences across pairs, and how to engage siblings in the promotion of PrEP.

## Results

### Quantitative Survey

Tables 1 and 2 provide descriptions of Latino SMM and their siblings. We enrolled 31 sibling dyads. The majority of sibling pairs (*n* = 18, 58%) did not reside together, and most of the siblings were older than the Latino SMM (*n* = 22, 35%). Almost all Latino SMM (*n* = 30; 96%) were open about their sexual orientation with their mother, while a smaller proportion had disclosed to their father (*n* = 21; 68%). Among Latino SMM, there was variation in their level of concern for HIV, with nearly 80% expressing a high level of concern. The majority of Latino SMM demonstrated awareness of PrEP (*n* = 27; 87%). However, only a small fraction felt very confident about where to access it (*n* = 10; 32%), and most participants were undecided about using PrEP (*n* = 17; 55%). Interestingly, more than 70% (*n* = 22) of participants reported that they would consider PrEP to alleviate their siblings’ concerns about HIV (Table 1).

**Table 1:** Descriptive statistics for siblings

	*n*	*%*
*Age* (in years)		
18–20	6	19
21–30	17	55
31–40	6	19
40 +	2	6
*Relative age to Latino SMM*		
Younger	20	65
Older	11	35
*Educational attainment*		
Some High School	2	6
High School	10	32
Some College	8	26
College	8	26
Professional School	3	10
*Brother’s sexual orientation disclosure*		
He told me	15	48
I found out	16	52
*Country of birth*		
Mexico	6	19
El Salvador	2	6
Guatemala	1	4
USA	22	71
*Aware of PrEP prior to study*		
No	13	42
Yes	18	58
*PrEP availability for my brother*		
Strongly disagree	4	13
Disagree	3	10
Unsure	9	29
Agree	8	26
Strongly agree	7	23
*Brother is at risk of HIV*		
Strongly disagree	7	23
Disagree	8	26
Unsure	9	29
Agree	7	23
Strongly agree	0	0
*Siblings’ HIV concerns: PrEP assurance*		
Strongly disagree	1	3
Disagree	1	3
Unsure	4	13
Agree	17	55
Strongly agree	8	26
*Support brother to use PrEP*		
Strongly disagree	1	3
Disagree	0	0
Undecided	6	19
Agree	12	39
Strongly agree	12	39
*Support brother to use PrEP by*		
Going to the doctor with him	28	90
Assist with refill	28	90
Assist with initial side-effects	28	90
Pill reminder	28	90

**Table 2:** Descriptive statistics for Latino men who have sex with men

	*n*	*%*
*Age*		
18–20	4	13
21–30	19	62
31–40	8	25
*Educational attainment*		
Some High School	1	4
High School	9	29
Some College	10	32
College	11	35
*Romantic relationship*		
No	20	65
Yes	11	35
*Live with sibling enrolled in study*		
No	15	48
Yes	16	52
*Country of birth*		
Mexico	8	25
El Salvador	2	6
Guatemala	1	4
USA	20	65
*Sexual orientation disclosure (Father)*		
Not out to father	10	32
Out to father	21	68
*Sexual orientation disclosure (Mother)*		
Not out to mother	1	4
Out to mother	30	96
*Concern for HIV*		
Strongly disagree	1	4
Disagree	2	6
Undecided	4	13
Agree	8	26
Strongly agree	16	52
*Aware of PrEP prior to study*		
No	4	13
Yes	27	87
*Access to PrEP*		
Strongly disagree	4	13
Disagree	6	19
Unsure	7	23
Agree	9	29
Strongly agree	5	16
*PrEP willingness*		
Disagree	1	3
Undecided	17	55
Agree	3	10
Strongly agree	10	32
*Consider using PrEP to ease sibling(s) HIV concern*		
Strongly disagree	1	4
Disagree	3	10
Undecided	5	16
Agree	10	32
Strongly agree	12	39

Most siblings reported that they found out about their brothers’ sexual orientation and/or behavior before their brothers formally came out to them (*n* = 16; 52%). Nearly all siblings (*n* = 25; 81%) reported strong concern for their brother and his risk of getting HIV. We found that 13 (42%) siblings were not aware of PrEP beforehand. Furthermore, a significant number of participants (*n* = 16; 52%) reported that their brothers had limited access to PrEP or lacked knowledge on how to obtain it. The majority of siblings (*n* = 24; 78%) indicated that they were willing to support their brother’s decision to use PrEP. Nearly all participants (*n* = 28; 90%) expressed their commitment to accompany their brother to the doctor, help with prescription refills and managing initial side effects, as well as reminding them to take PrEP (Table 2).

### Qualitative Overview

The interviews revealed several key themes and categories, encompassing: (1) Sibling support for coming out; (2) Types of support siblings provide to each other for behavior change; (3) Sibling support for PrEP; and (4) Impact of the study interview on the quality of the sibling relationships. In the forthcoming analysis of each theme and the corresponding firsthand responses, participants have been assigned pseudonyms to ensure anonymity. Additional quotes for each theme are provided in Tables 3 and 4, sources from siblings and Latino SMM, respectively.

**Table 3:** Exemplary quotes from siblings

Theme	Demonstrative quote
Sibling support to coming out	Our mom did not understand when he came out, but with me, he is my brother, and he is never going to have to worry about not having support, because I am going to be there. Even when he was younger and had more feminine interests, I would be there to embrace them, so that he felt supported.
Type of support siblings provide to each other for behavior change	He has always been a motivation to me. I get to see how much he has flourished as an undocumented student not knowing English at such a young age. Just seeing that persistence helped me want to succeed at school or at work.
Sibling support for PrEP	I would put notes on the door or next to his wallet, so that he would see it before going to work. I would also be annoying and maybe leave a trace of pills around the house so that he would see them and be able to take them right away.
Impact of the STudy interview	At the end of the day he is my best friend, and it was nice to be able to talk to him about PrEP, so that he can trust me.

**Table 4:** Exemplary quotes from Latino SMM

Theme	Demonstrative quote
Sibling support to coming out	My sister would bring around her gay friends, so when I told her I was gay, it was so natural. We would stay up late and talk about boys, and that made it seem so normal for me.
Type of support siblings provide to each other for behavior change	When there is an issue at home or with our family, I have asked him for his advice on how to talk to our parents. He is honest with me, and that is sometimes what I need, but I guess he also helps me out with planning finances and with working out.
Sibling support for PrEP	I guess since we are both gay, we could remind each other, or even share which doctor we liked more.
Impact of the study interview	I thought talking to my brother who is straight about PrEP would be uncomfortable, but it wasn’t. I can see how helpful it was to have this conversation with him.

### Sibling Support for Coming Out

Descriptions of sibling support following Latino SMM’s coming out were significant in the narratives of all participants and their siblings. Not surprisingly, many siblings expressed their support when their brother came out. For example, Anthony, an older heterosexual brother to Homero, shared:

Personally, it wasn’t a big deal to me when he came out because he’s still my brother. It doesn’t change who he is as a person. So, that was that. It wasn’t a major issue, but I’m glad he felt comfortable telling me.

Although several siblings were not surprised when their brother came out to them or when they learned about it through someone else, they did express concern about how their parents, given their cultural and ethnic background, would react to their brother’s disclosure. Yamilet, an older heterosexual sister to Steven, stated, “I didn’t want him to be gay because I know our family is very religious and not open minded. They believe a boy should be with a girl, you know. So that was my fear.” Other siblings also shared their apprehensions about their families’ potential reactions to their brother coming out given their family’s conventional perspective on masculinity and gender. Rubi, an older heterosexual sister to Alfonso, shared how they would express their mutual worry about their father’s potential reaction. She stated:

My brother would cry when we would talk about him being gay. He didn’t want to tell our family, he didn’t want to accept it. “But Rubi,” he would say, “I can’t–my dad, rest in peace, he wanted me to be a man like he wanted me to be, and our siblings, what are our siblings going to think? The family?” He was a person who was very–I mean, our family was very rooted in male chauvinism. So, we talked, and we talked, and I understood him.

Importantly, many Latino SMM emphasized the significant role their siblings played in normalizing their journey of coming out, providing a safe space for open communication about their sexuality. These conversations held immense importance, particularly because siblings often served as the sole source of support within their families. This support proved even more crucial for those Latino SMM who had fears about coming out to their parents. For instance, Rubi, who is Alfonso’s older heterosexual sister, shared, “Initially, everyone rejected him, and he gave me permission to talk to our family to help them be more accepting. I opened a path for them to talk with him.” Furthermore, these siblings not only offered emotional support when their brothers came out, but also actively advocated for them when needed or when approached by their parents. For example, Daisy, who is Robert’s older heterosexual sister, shared that when Robert came out to their father and faced anger and lack of support, she stood up for him, playing a pivotal role in fostering their father’s understanding and acceptance.

In interviews where participants were accompanied by their gay brothers, some expressed how their brothers played a crucial role in facilitating their own coming out process. This was particularly evident in situations where their sibling had already come out themselves. Joshua, an older gay brother to Aaron, spoke about how his brother’s disclosure made it easier for him [Joshua] to come out to their parents. He stated, “My brother encouraged me that it would be better to tell [mom] so that she could stand up more to our father, so I told her.” These findings were corroborated by participants who experienced an improved bond with their sibling as they discovered their shared identity as gay individuals. This newfound connection fostered a deeper sense of closeness, particularly for those who had previously harbored suspicions about their sibling’s sexual orientation. Juan’s older gay brother, Joel, mentioned, “I knew that my brother was gay because I myself was gay, but I didn’t know. So, I felt relieved when he told me because we connect on a different level now*.*”

### Types of Support Siblings Provide for Behavior Change

Siblings often serve as positive role models and sources of support for their brothers. Older siblings in particular discussed the ways in which they provided emotional support to help their brother reduce harmful behaviors. Kelly, a heterosexual Latina, and older sister to Brandon, expressed her concern when she observed her brother’s increased socializing and drinking habits after he came out. In response, Brandon acknowledged that Kelly’s words impacted him: he reduced his drinking. He said:

Having conversations with her changed my way of thinking and ways of doing things. She would always tell me to wake up and to not do something stupid in the street while I am drinking because I could end up doing something scary.

Similarly, David highlighted the valuable role played by his older heterosexual sister, Monica, in providing crucial support to address his drinking habits. This conversation, which he found challenging to have with his parents, ultimately resulted in a positive change in his alcohol consumption. He noted:

I became that party guy. I just wanted to go out and drink. And she’s always been there to snap some sense into me back. She would tell me, “You need to get back on track. You need to focus on what you’re doing. You need to not drink as much,” and stuff like that. So, I feel she’s always been very supportive in that way where she can give me a reality check that I can’t have as easily with my mom.

As previously mentioned, siblings who are themselves gay can provide invaluable support for behavior change due to their shared identities. For instance, RK recounted how his older brother Jay offered guidance when he desired to socialize more frequently. However, Jay also advised him that constant outings were not necessary, despite the initial perception. This counsel ultimately had a positive impact on RK’s behavior, as Jay was able to draw from his own experiences in the queer community and the mistakes he made under the pressure to socialize and drink excessively. Several participants reported that they were influenced by observing their sibling’s healthy habits, which led to changes in their own behavior. For instance, Coki, John’s younger sister, mentioned that her brother inspired him to be more mindful of taking daily vitamins and making other health adjustments. This positive influence was reciprocal, as Coki also shared that once he embarked on his journey to quit drinking, John was likewise motivated to quit.

Some sibling pairs confronted socioeconomic hurdles during their early years, including experiencing homelessness and limited health insurance coverages. These shared hardships significantly shaped the mutual support they would later extend to each other, encouraging healthier habits and lifestyle choices. As an example, Marie, the older heterosexual sister of Slayvin, recounted memories of relying on her brother for emotional strength during her challenging experience in the foster system at the age of 15. She stated.

He would help me change my behaviors by calling me and telling me it’s not worth it to run away from my placement and just his conversations towards me about not running away from my problems, helped me stay there and get over them instead of running away from them.

In a similar vein, numerous siblings and their brothers engaged in discussions about how they could mutually support each other in setting new goals, whether they be related to health or education. For instance, Cristina, Teddy’s younger heterosexual sister, shared her plans to encourage him to embrace greater challenges and set more ambitious goals. Teddy expressed his gratitude, acknowledging her unwavering willingness to help him overcome any obstacles.

During their conversations, many participants highlighted their comfort in openly discussing their romantic and sexual relationships with their siblings. For example, some sibling pairs shared how they were able to have honest conversations with each other about engaging in sexual activity without protection with their respective partners. According to Naw, Leonardo’s older heterosexual brother, they had a code word to refer to having unprotected sex with someone—they called it “BB’d” [barebacked]. This was their way of communicating about sexual relationships. Similarly, Kristy, the older heterosexual sister of Christian, revealed that Christian would openly share his Grindr profile with her. He would also discuss the details of his sexual encounters, prompting Kristy to advise him to get tested and even suggest which clinics to visit. These discussions, irrespective of the gender or sexual orientation of their siblings, brought about a normalization of sex-related health topics. This was particularly valuable as many participants felt unable to have such conversations with their parents due to their cultural background.

### Sibling Support for PrEP

Participants were surveyed regarding their knowledge of PrEP for HIV prevention and their potential support for their brothers in using PrEP. Out of the 31 siblings, only 13 were aware of PrEP. However, once informed, nearly all of them expressed their support for their brother’s use of PrEP. They also engaged in discussions on how to encourage adherence and ensure consistent use of PrEP, whether in the form of a daily pill or through injections. For instance, Liz, Ches’s older heterosexual sister, mentioned that she would remind him to adhere to the daily PrEP regimen or follow through with the injections if he opted for long-acting injectable PrEP. She further emphasized, “Personally, I would be there to accompany him to the clinic if he needed transportation. I would be with him.”

In response to Latino SMM’s uncertainty about potential side effects of PrEP, some siblings shared their commitment to supporting their brothers and alleviating their stress, offering unconditional support. Marie, the older heterosexual sister of Slayvin, expressed her willingness to support him and serve as a valuable resource. Recognizing her brother’s uncertainty, she offered herself as a trusted confidant to help him make an informed decision. She stated:

I would honestly love to talk to my brother about PrEP because I feel like it would be good for him to express how he feels about the medication and about his concerns taking it, instead of just being undecisive and not having no one to talk about it.

Other Latino SMM expressed concern over how their use of PrEP would be seen by others. In their feedback, siblings expressed concern over their brothers’ risk of getting HIV and emphasized PrEP as a respectable form of self-care. For example, Cristina, Teddy’s younger heterosexual sister, acknowledged that while she initially had reservations about PrEP, she recognized that PrEP is a means to ensure his safety, particularly considering that not everyone may exercise the same level of caution as he does. She stated: “As your sister, I would tell you to use PrEP. Because again, it’s a crazy world out there, and if you have that extra layer of protection, there’s no harm in using it.”

Notably, during the interviews, it was common for siblings to discuss various ways they would support their brother in using and adhering to PrEP. Many mentioned instrumental support, such as reminding their brother to take the pill through regular check-ins or sending reminder text messages. Some even expressed their willingness to assist their brother in finding a suitable clinic and offering to accompany or transport them if necessary. For example, Stiles, younger heterosexual brother to Adrian, stated: “I would suggest he put an alarm on his phone every day. Not only that but say around 12:00 I would remind him to take his pill.” As previously stated, among the 31 siblings who took part in our study, 18 of them were already familiar with PrEP before their interviews. Out of these 18 individuals, 6 siblings shared during their interviews that they had already recommended PrEP to their brothers after learning about it. Kristy, older heterosexual sister to Christian, noted: “When I first heard about PrEP [before participating in the study], I was like, ‘Hey, there’s thing called PrEP to prevent HIV. You should look into it’.”

### Impact of the Study Interview on Sibling Relationships

Although our study did not specifically examine the impact of discussing PrEP with siblings on their relationship or their decision to use PrEP, numerous participants expressed feeling a stronger bond with their siblings and a greater sense of willingness to use PrEP after participating in the dyadic interviews. Participants shared their surprise upon learning about the extent of their siblings’ concern for their sexual health. Moreover, they were pleasantly surprised by the level of support their siblings provided for their sexual orientation and relationships. For instance, one participant stated: “I feel like it’s a good thing that we’re both talking about it. I feel like I can talk to her even more [about] stuff now, since we’re talking about sex [now]. So, it’s a good thing that we did today.”

Furthermore, participants expressed the significance of engaging in such a dialogue with their sibling and experiencing acceptance from a close family member. Junior had a strained relationship with his older heterosexual brother, Lalo, when he first came out over 10 years ago. Although they now had a good relationship, Junior did not realize just how much his brother had changed. Junior said:

I was about to start crying as I was hearing him talk about how he would support me to use PrEP. I don’t know, it feels very good to hear that I am being accepted. Like from not being accepted to being accepted, it feels like it is gold, man. It’s really something that not a lot of people get, so I feel very fortunate and lucky and blessed that he accepted me.

In the end, while a few participants initially felt uneasy discussing PrEP and their sexual health with their siblings present, they gradually grew more comfortable by the end of the interview. Some even expressed a newfound confidence in their ability to engage in similar conversations in the future, free from the fear of being judged or rejected. Even Latino SMM who were hesitant to talk about sexual health with their brother expressed a changed in perspective and willingness to share more with their sibling after participating in the study. Adrian, younger heterosexual brother to Stiles, captures this sentiment: “I think I felt a little uncomfortable. But at the same time, you have to have these uncomfortable conversations sometimes. Especially with your family, because I think those are the people who you can trust the most, or you should be able to feel free to be able to communicate [to] them whatever it is that you’re going through. I think it’ll be more normal from now on, to have these conversations.”

## Discussion

The literature consistently emphasizes the importance of cultural factors, such as familial relationships, societal reluctance to discuss sexuality, and traditional gender norms, in the design and implementation of HIV prevention programs for Latino populations. These cultural factors have also been prioritized by the CDC. However, only a few studies have directly incorporated these cultural factors into their programs (Lescano et al., 2009; Wilson & Miller, 2003; Wyatt, 2009). Our study explored the potential role of siblings in promoting the use of PrEP for HIV prevention. This is particularly noteworthy as most research on conversations about sexual health and HIV with adolescents has focused on the parent–adolescent relationship, thus setting a precedent for incorporating siblings into prevention interventions (Grossman et al., 2015, 2020).

While previous research has almost exclusively focused on the ways in which older siblings generally increase and/or decrease sexual risky behaviors in younger siblings (Averett et al., 2011; Coleman-Minehan & Scandlyn, 2017; Pasqualini et al., 2021), less research has focused on the type of relationships between Latino SMM and their siblings. Less is even understood, related to sexual health and the mechanisms through which siblings could support HIV prevention among this population. Based on our data, siblings, regardless of their gender or sexual orientation, viewed PrEP as an effective and appropriate method to protect their brothers from HIV transmission. This held true even when they had concerns about potential side effects or their families’ reactions. These findings indicate that while family dynamics and socio-cultural factors may present obstacles to HIV prevention among certain Latinos (Ramírez-Ortiz et al., 2020), they can also serve as sources of support when further explored. These findings align with studies demonstrating that Latino SMM often reconcile with their families over time despite initial experiences of rejection, and often rely on mutual support from their siblings as they age (Abreu et al., 2020; Cahil et al., 2021).

The theme of Support for Coming Out encompasses the significance of sibling relationships in adolescents’ lives, as well as the shared aspects of identity, experiences, and characteristics between adolescents and their siblings. Participants emphasized the pivotal role their siblings played when they first came out, highlighting how this support alleviated distress during this crucial developmental phase. These findings align with previous research on sexual minority adolescents, which underscores the importance of sibling support during the coming out process (Haxhe et al., 2018; Mathers, 2019). Furthermore, the parallel between the current findings and research on HIV prevention suggests that siblings may be more inclined to support and be receptive to PrEP due to their shared experiences and prior support during the coming out process. In contrast, previous research suggests that parents, influenced by stronger cultural ties to traditional Latino culture, may exhibit less support toward sexual identity (Gattamorta et al., 2019) or, in the context of our study, PrEP.

The theme Types of Support Siblings Provide for Behavior Change offers an overview of various forms of support, both formal and informal, that Latino SMM and their siblings offer to one another to foster healthier behaviors using strategies such as encouragement and role modeling. Many Latino SMM and their siblings, for example, discussed openly the ways in which they would encourage each other to drink less and more responsibly, often also suggesting other ways to cope and providing additional resources. Several siblings also shared how they encouraged healthier eating habits and physical activity in their brothers, and vice versa. They recognized the positive impact of their role modeling in shaping these behaviors. Notably, these findings shed light on the positive dynamics of sibling support, expanding upon prior research (Cardoza et al., 2012; Grossman et al., 2015, 2020; Stauss et al., 2011; Whiteman et al., 2014). While previous research has predominantly examined how families among Latinos can negatively impact the health of sexual minorities (Ryan et al., 2009), our study offers valuable evidence on the specific ways in which siblings play a supportive role in fostering various adaptive health behaviors.

The theme Sibling Support for PrEP offers insights into how Latino SMMs siblings might play a crucial role in promoting and ensuring the successful uptake and adherence to PrEP for HIV prevention. Almost all Latino SMM expressed some degree of familiarity with PrEP, but none actually had been prescribed PrEP. In their stories, Latino SMM highlighted obstacles to PrEP involvement, which aligns with previous research. These obstacles included concerns about potential side effects, the high cost of medication, and limited social support (Zapata et al., 2023). Upon being provided with factual information related to PrEP, nearly all of the siblings in our study expressed their support of PrEP for their respective brothers to use as a way to prevent HIV infection, with many being surprised that they had not heard of PrEP before. Across each interview, siblings offered their support for PrEP, ranging from emotional support if their brothers were concerned about potential rejection to more logistical support, such as providing transportation or assisting with costs. While previous research has primarily concentrated on the concerns of Latino SMM regarding their families’ lack of support for PrEP usage (Zapata et al., 2023), these findings represent one of the initial attempts to document the unwavering support of Latino siblings for PrEP use, irrespective of their gender or sexual orientation. As such, siblings can be a critical resource for information and support when it comes to PrEP uptake among Latino SMM.

In addition to understanding the extent to which siblings provide unwavering support for PrEP usage, it is also important to understand how culturally specific socialization practices can shape their reactions and beliefs (Updergraff et al., 2005). For example, many of the participants in our study reported that their siblings were unified and strived to protect one another, which is a common theme in Latino cultures (Streit et al., 2018). By offering to provide unwavering support for PrEP usage among SMM, these siblings are also upholding traditional values of family unity and care within the Latino context. These findings offer a promising outlook on how Latino families can contribute to HIV prevention efforts.

While further research is warranted, our findings suggest that siblings can serve as a valuable resource to enhance PrEP acceptability within this population. Additionally, this can raise awareness of PrEP among Latina women, who have been largely underrepresented in research and prevention efforts (Nieto et al., 2020). These findings were further supported by our exploratory findings, where we discovered that when discussing PrEP with their brothers, some siblings expressed their own interest in learning more about PrEP as a viable option for themselves. They also requested additional clinical resources to aid in their understanding.

### Limitations, Future Directions, and Implications

This study is limited by its convenience sample, which disproportionately includes Latino SMM who are open to their families about their sexual orientation and who share a close relationship with at least one sibling. However, given the lack of studies on the relationship Latino SMM have with their siblings and their coming out process, and how siblings might support HIV prevention efforts, these findings provide a much-needed start to understanding family dynamics in this population. Future research would benefit from including multiple perspectives on extended family relationships, such as with cousins, given that not all Latino SMM have siblings of their own or if they do, some might not be out to them (Caba et al., 2022), to identify other familial avenues through which to support PrEP and sexual health communication. Additionally, our sample size was limited, and further research should be conducted to identify if these findings are generalizable to larger Latino populations. Furthermore, although the researchers who conducted the semi-structured interviews and coded the data were themselves Latino and sexual minority men, the qualitative results are influenced by the subjectivity inherent in interviews, which impacts the identified themes in the study. However, to mitigate this risk, multiple coders were involved, and the findings were discussed with other researchers who were not part of the coding process.

Furthermore, given that the majority of participants were of Mexican heritage, it is imperative to conduct additional research with other Latino ethnic groups in the USA. This will help us understand the potential variations in sexual health communication within different Latino communities. However, it is likely that, similar to previous research on Latinos, disparities in sexual health communication are influenced by factors such as acculturation and generational differences, rather than specific sub-ethnic distinctions (Cervantes et al., 2019). Due to the limited sample size, it was not possible to compare the responses of sibling dyads in terms of age or sexual orientation. Additionally, we did not collect specific ages for the Latino SMM or their siblings, so our results only include age ranges. However, this limitation can be addressed in future studies to further explore these aspects. Furthermore, this study did not capture the dynamic nature of sibling relationships. Future studies should investigate how siblings’ roles evolve over time and if these changes alter family support for SMM health-related decisions. Additionally, it is crucial for future studies to consider potential barriers that may arise among Latino SMM living with their siblings, especially in light of reported challenges related to PrEP among SMM living with their families. This could include addressing concerns such as medication security and providing guidance on how to discuss PrEP in a non-stigmatizing manner with their families.

Our findings have implications for the design of HIV prevention interventions for Latinos. These interventions should consider incorporating siblings or other extended family members to facilitate communication between siblings and their brothers, encouraging the use of PrEP or similar prevention methods. Additionally, our findings suggest that strengthening sibling relationships and fostering openness can have additional benefits in supporting various health-related behaviors, including reduced drinking and increased physical exercise. Moreover, our results highlight the safeguarding role that Latino siblings may play in supporting their brothers during the process of coming out, which represents an unexplored avenue of support that could alleviate the detrimental consequences of minority stress among Latino SMM (Shramko et al., 2018; Zelaya et al., 2023). Finally, as the CDC continues to promote the dissemination of PrEP to communities at risk of HIV infection, involving siblings through various intervention channels has the potential to improve access and uptake of PrEP among Latino women and other non-sexual minority individuals. This approach could also help destigmatize PrEP usage across communities, by presenting it as an HIV prevention method that is not exclusive to men who have sex with men (Calabrese & Underhill, 2015).

## Data Availability

Data are not accessible to the public to minimize the risk of loss of confidentiality. Data are available upon request to the Administrative Core of the Health Intervention Sciences Group / Center for AIDS Intervention Research. Individuals who meet criteria for access to de-identified data should contact the Principal Investigator.
